# Inhibition of ribosome assembly factor PNO1 by CRISPR/Cas9 technique suppresses lung adenocarcinoma and Notch pathway: Clinical application

**DOI:** 10.1111/jcmm.17657

**Published:** 2023-01-10

**Authors:** Sanjit K. Roy, Shivam Srivastava, Andrew Hancock, Anju Shrivastava, Jason Morvant, Sharmila Shankar, Rakesh K. Srivastava

**Affiliations:** ^1^ Louisiana State University Health‐New Orleans, School of Medicine Stanley S. Scott Cancer Center New Orleans Louisiana USA; ^2^ Southeast Louisiana Veterans Health Care System New Orleans Louisiana USA; ^3^ Holy Cross New Orleans Louisiana USA; ^4^ Department of Molecular and Cellular Biology Tulane University New Orleans Louisiana USA; ^5^ St. Joseph's Hospital and Medical Center Phoenix Arizona USA; ^6^ Department of Surgery Ochsner Health System Gretna Louisiana USA; ^7^ Department of Genetics Louisiana State University Health Sciences Center New Orleans Louisiana USA; ^8^ John W. Deming Department of Medicine Tulane University School of Medicine New Orleans Louisiana USA; ^9^ Kansas City VA Medical Center Kansas City Missouri USA

**Keywords:** biogenesis, CRISPR/Cas9, EMT, lung adenocarcinoma, Notch, PNO1

## Abstract

Growth is crucially controlled by the functional ribosomes available in cells. To meet the enhanced energy demand, cancer cells re‐wire and increase their ribosome biogenesis. The RNA‐binding protein PNO1, a ribosome assembly factor, plays an essential role in ribosome biogenesis. The purpose of this study was to examine whether PNO1 can be used as a biomarker for lung adenocarcinoma and also examine the molecular mechanisms by which PNO1 knockdown by CRISPR/Cas9 inhibited growth and epithelial–mesenchymal transition (EMT). The expression of PNO1 was significantly higher in lung adenocarcinoma compared to normal lung tissues. PNO1 expression in lung adenocarcinoma patients increased with stage, nodal metastasis, and smoking. Lung adenocarcinoma tissues from males expressed higher PNO1 than those from females. Furthermore, lung adenocarcinoma tissues with mutant Tp53 expressed higher PNO1 than those with wild‐type Tp53, suggesting the influence of Tp53 status on PNO1 expression. PNO1 knockdown inhibited cell viability, colony formation, and EMT, and induced apoptosis. Since dysregulated signalling through the Notch receptors promotes lung adenocarcinoma, we measured the effects of PNO1 inhibition on the Notch pathway. PNO1 knockdown inhibited Notch signalling by suppressing the expression of Notch receptors, their ligands, and downstream targets. PNO1 knockdown also suppressed CCND1, p21, PTGS‐2, IL‐1α, IL‐8, and CXCL‐8 genes. Overall, our data suggest that PNO1 can be used as a diagnostic biomarker, and also can be an attractive therapeutic target for the treatment of lung adenocarcinoma.

## 1 INTRODUCTION

Lung cancer is the third most common cancer in the United States of America. According to the American Cancer Society's estimates, 236,740 new cases of lung cancer (117,910 in men and 118,830 in women) will be diagnosed and 130,180 deaths from lung cancer (68,820 in men and 61,360 in women) will occur in 2022.^1^ Generally, in a vast majority of patients, lung cancer is metastasized and therefore it becomes incurable.^2^ Despite recent advances in lung cancer, the 5‐year survival rate of patients with lung cancer is 16%.^1^ Therefore, it is urgent to identify new molecular biomarkers to predict the prognosis of lung cancer patients and develop novel molecular targeted therapy for lung adenocarcinoma.

The ribosome, conserved from yeast to mammals, is a ribonucleoprotein complex that regulates translation machinery during protein synthesis.^3^, ^4^ The higher energy demand of cancer cells is associated with increased ribosome biogenesis and mutation of ribosomal proteins. Therefore, ribosome represents an attractive anti‐cancer therapy target. The RNA‐binding protein “partner of NOB1” (PNO1, also known as Dim2, Rrp20) is a ribosome assembly factor.^3^, ^5^ The PNO1 gene is located in human chromosome 2p14, comprising seven exons and six introns, and plays a crucial role in ribosome biogenesis and promotes the maturation of small ribosomal subunits.^5^ PNO1 cleaves 18S mediated by binding to NOB1.^6^ In spite of its role in ribosome biogenesis, the mechanism by which PNO1 regulates oncogenesis is not well understood. Since PNO1 is highly expressed in cancer cells,^7^, ^8^, ^9^, ^10^, ^11^ it can be used as a diagnostic biomarker and also can be an attractive target for cancer therapy. Therefore, understanding the expression and biological function of PNO1 is crucial for effectively managing lung adenocarcinoma.

Cancer cells generally possess unlimited replicative potential, divide faster, and display increased biosynthesis and metabolic activity to meet enhanced energy demand.^12^ Thus, frequently dividing cancer cells will require enhanced global protein synthesis. One of the mechanisms by which protein synthesis can be controlled through an increase in mRNA translation requires ribosomes.^13^ Ribosomes are produced in the nucleolus and act as molecular machines where they translate mRNA into protein. Recent studies have demonstrated the dysregulated ribosome biogenesis during carcinogenesis.^14^, ^15^ Increased size and numbers of nucleoli are frequently observed in most cancers, requiring malignant cells to acquire enhanced ribosomal biogenesis. This suggests that increased ribosome biogenesis may play an essential role in cancer initiation and progression. Therefore, inhibition of ribosome biogenesis may provide an attractive therapeutic strategy for the treatment of cancer. To address this, we have inhibited the expression of PNO1 by CRISPR/Cas9 technology and examined the inhibitory effects of PNO1 on the mechanism of lung carcinogenesis.

The notch pathway is highly conserved and mediates cell‐to‐cell interaction in eucaryotes.^16^, ^17^, ^18^ The notch pathway regulates cell growth, development, differentiation, and apoptosis.^16^, ^19^, ^20^, ^21^ The Notch ligands are the single‐pass transmembrane proteins of the DSL family, consisting of five ligands namely, Jagged‐1, Jagged‐2, Delta‐like‐1 (Dll1), Delta‐like‐3 (Dll‐3), and Delta‐Like‐4 (Dll‐4). There are four Notch receptors, referred to as Notch‐1, Notch‐2, Notch‐3, and Notch‐4.^19^, ^22^ The Notch receptors are transmembrane proteins consisting of extracellular and intracellular domains.^19^ The binding of ligand to the receptor causes a conformational change leading to ADAM‐mediated ectodomain shedding and subsequent γ‐secretase‐mediated proteolysis within the transmembrane domain, resulting in the release of Notch intracellular domain (NICD).^23^ Subsequently, NICD translocates to the nucleus and associates with the RBPjκ to form an active protein complex (Mastermind, histone acetyltransferase, and p300), leading to the induction of target genes such as Hes1 and Hey1. The oncogenic role of Notch in lung cancer has been demonstrated.^24^, ^25^, ^26^ Therefore, inhibition of the Notch signalling pathway represents a novel therapeutic strategy for managing lung adenocarcinoma.

The main objective of this paper is to assess whether PNO1 can be used as a diagnostic marker for lung cancer and examine the molecular mechanisms by which PNO1 inhibition regulates lung cancer growth and epithelial–mesenchymal transition (EMT) in lung cancer. The expression of PNO1 was significantly higher in lung cancer cells than in adjacent normal tissue, suggesting it can be used as a diagnostic marker for lung cancer. PNO1/CRISPR/Cas9 inhibited growth and EMT of lung cancer cells by suppressing the Notch pathway. PNO1 knockdown inhibited those genes which play significant roles in cell proliferation, cell cycle, apoptosis, and EMT. In addition, PNO1/CRISPR/Cas9 also inhibited Notch signalling pathway, which plays a crucial role in lung carcinogenesis. In conclusion, PNO1 can be used as a diagnostic biomarker for lung adenocarcinoma, and inhibition of PNO1 by CRISPR/Cas9 technology can be a useful strategy for the treatment of lung adenocarcinoma.

## 2 MATERIALS AND METHODS

### 2.1 Reagents

Fetal bovine serum, penicillin, Matrigel, and streptomycin were purchased from Thermo Fisher Scientific (Suwanee, GA). All other chemicals were purchased from Sigma–Aldrich (St. Louis, MO). PNO1 lentiviral vectors containing either non‐targeting control (NTC) or PNO1/CRISPR/Cas9 were purchased from Gene Copoeia (Rockville, MD).

### 2.2 Cell culture

Human lung cancer cells (A549 and H460) were purchased from American Type Culture Collection (ATCC, Manassas, VA). A549 and H460 cell lines both express wild‐type p53.^27^, ^28^ Lung cancer cells were grown in RPMI 1640 culture medium containing 10% fetal bovine serum, 100 U/ml penicillin, and 100 μg/ml streptomycin at 37°C in a humidified atmosphere of 95% air and 5% CO_2_.

### 2.3 Cell viability assay

Cell viability was measured as described elsewhere.^29^ In brief, cells (1.5 × 10^4^) transduced with NTC or CRISPR/Cas9 were grown in a cell culture medium for various time points. Cell viability was measured by CellTiter‐Glo® Luminescent Cell Viability Assay (Promega), which determines the number of viable cells based on the quantitation of the ATP present, which signals the presence of metabolically active cells.

### 2.4 Apoptosis assays

Apoptosis was measured as described elsewhere.^30^ In brief, cells transduced with either NTC or CRISPR/Cas9 lentiviral particles were grown in a cell culture medium at various times. Apoptosis was measured by TUNEL assay as per manufacturer's instructions (Thermo Fisher Scientific, Suwanee, GA).

### 2.5 Motility assay

Cell motility assay was performed as we described elsewhere.^31^, ^32^ In brief, cells (NTC and CRISPR/Cas9) were grown to a confluent monolayer in a 6‐well plate, scratched with a 200‐μl tip, and washed twice with PBS. Lung cancer cells were grown in RPMI1640 and photographed at 0 and 48 h under an inverted microscope at 40× magnification. We viewed the width of the scratch gap under the inverted microscope until the gap was filled in the untreated control wells.

### 2.6 Transwell migration assay

Transwell migration assays were performed as we described elsewhere.^33^ In brief, 1 × 10^5^ cells (NTC and CRISPR/Cas9) in 200 μl of medium with 1% FBS were plated in the top chamber onto the non‐coated membrane (6.5‐mm diameter, 8‐μm pores; Corning Costar, Corning, NY) and allowed to migrate in the lower chamber towards 10% FBS (as chemoattractant)‐containing medium. After 48 h of incubation at 37°C in 5% CO_2_, we fixed the cells with methanol, stained with crystal violet, and counted under an inverted microscope.

### 2.7 Transwell invasion assay

Transwell invasion assays were performed as we described elsewhere.^34^, ^35^ In brief, 1 × 10^5^ cells (NTC and CRISPR/Cas9) in 200 μl of medium with 1% FBS were plated on top of a layer of Matrigel in transwell chambers (6.5‐mm diameter, 8‐μm pores; Corning Costar, Corning, NY). We allowed the cells to invade the lower chamber towards 10% FBS (as chemoattractant)‐containing medium. After 48 h of incubation at 37°C in 5% CO_2_, cells that did not migrate were removed from the top of the transwell filters by scraping. Cells in the lower chamber that had penetrated the Matrigel were fixed with methanol, stained with crystal violet, and counted under an inverted microscope. The invasion activity refers to the number of penetrated cells.

### 2.8 Western blot analysis

Western blot analysis was performed as we described elsewhere.^36^ In brief, cell lysates were prepared using RIPA lysis buffer containing 1 X protease inhibitor cocktail (Sigma–Aldrich, St. Louis, MO). Cell lysates containing 40–60 μg of protein were loaded and separated on 10% Tris–HCl gel. Proteins from the gel were transferred on polyvinylidene difluoride (PVDF) membranes and subsequently blocked in blocking buffer [5% nonfat dry milk in 1 X Tris Buffer Saline (TBS)] and incubated overnight with primary antibodies (1:500 or 1:1000 dilution). Membranes were washed three times with TBS‐T for 10, 5, and 5 min each. After washing, membranes were incubated with secondary antibodies conjugated with horseradish peroxidase at 1:5000 dilution in TBS for 1 h at room temperature. Membranes were again washed three times in TBS‐T (5 min each time) and developed using ECL Substrate. Protein bands were visualized on X‐ray film using an enhanced chemiluminescence system.

### 2.9 Quantitative real‐time PCR

Total RNA was extracted from cells using the TRIzol reagent as per the manufacturer's instructions (Thermo Fisher Scientific, Suwanee, GA). After checking the RNA quality and concentration, qRT‐PCR was performed as described elsewhere.^32^, ^37^ Briefly, cDNA was synthesized using a high‐capacity cDNA reverse transcription kit (Applied Biosystems). Primers specific for each of the signalling molecules were designed using NCBI/Primer‐BLAST and used to generate the PCR products. For the quantification of gene amplification, qRT‐PCR was performed using an ABI 7300 Sequence Detection System in the presence of SYBR‐Green.

### 2.10 Statistical analysis

The mean and SD were calculated for each group of the experiment. Differences between groups were analysed by anova or *t*‐tests using PRISM statistical analysis software (GrafPad Software, Inc.). Significant differences among groups were considered at *p* < 0.05.

## 3 RESULTS

### 3.1 The Cancer Genome Atlas (TCGA) reveals the differential expression of PNO1 which increase with smoking in lung adenocarcinoma

**FIGURE 1:**
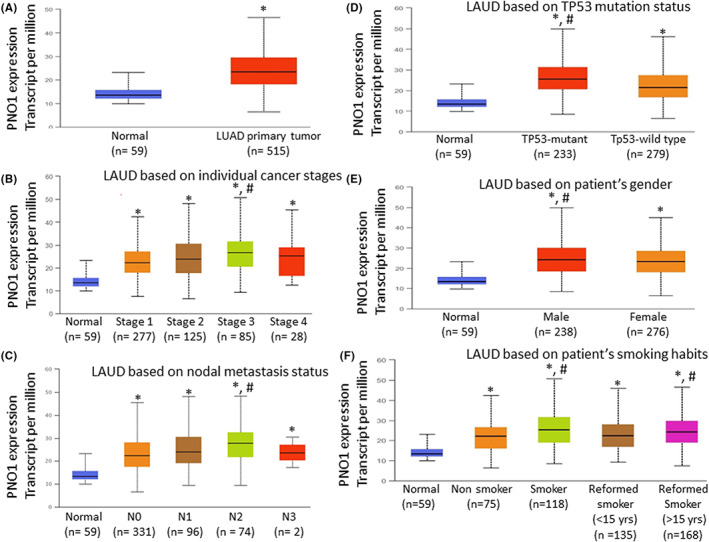
Differential expression of PNO1 increases with stage, nodal metastasis, and smoking in lung adenocarcinoma. (A) PNO1 expression in lung adenocarcinoma (LUAD) and normal lung tissues. PNO1 expression data were analysed using the Cancer Genome Atlas (TCGA) data bank by UALCAN (The University of Alabama at Birmingham Cancer Data Analysis Portal). Normal (*n* = 59), LUAD primary tumour (*n* = 515), * = significantly different from normal (*p* < 0.05). (B) PNO1 expression during various stages of lung adenocarcinoma. Normal tissues (*n* = 59), Stage 1 (*n* = 277), Stage 2 (*n* = 125), Stage 3 (*n* = 85), and Stage 4 (*n* = 28), *, # = significantly different from normal and each other (*p* < 0.05). (C) PNO1 expression at different stages of nodal metastasis. Normal (*n* = 59), N0 (*n* = 331), N1 (*n* = 96), N2 (*n* = 74), and N3 (*n* = 2), *, # = significantly different from normal and each other (*p* < 0.05). (D) PNO1 expression in lung adenocarcinoma with mutant and wild‐type Tp53 status. Normal (*n* = 59), Tp53 (*n* = 233), and Tp53‐wild‐type (*n* = 279), *, # = significantly different from normal and each other (*p* < 0.05). (E) PNO1 expression in male and female lung adenocarcinoma patients. Normal (*n* = 59), male (*n* = 238), female (*n* = 276), *, # = significantly different from normal and each other (*p* < 0.05). (F) Changes in PNO1 expression in LAUD with smoking. Normal (*n* = 59), Nonsmoker (*n* = 75), Smoker (*n* = 118), Reformed Smoker <15 years (*n* = 135), and Reformed Smoker >15 years (*n* = 168), *, # = significantly different from normal and each other (*p* < 0.05).

PNO1 has been associated with cancer progression and poor survival.^8^, ^9^, ^38^ We first examined the expression of PNO1 using the Cancer Genome Atlas (TCGA) data bank by UALCAN (The University of Alabama at Birmingham Cancer Data Analysis Portal). As shown in Figure 1A, the expression of PNO1 was significantly higher in lung adenocarcinoma than in normal lung tissues. We next examined whether the expression of PNO1 changed during various stages of lung adenocarcinoma (Figure 1B). PNO1 expression was significantly higher in all stages (stages 1–4) of lung adenocarcinoma development compared to normal tissues. The highest expression of PNO1 was observed in stage 3. We next examined the PNO1 expression at different stages of nodal metastasis. PNO1 expression was significantly higher in all stages (N0–N3) of nodal metastasis compared to normal tissues (Figure 1C). The highest expression of PNO1 was observed at N2 metastasis. We next examined whether Tp53 mutant status plays any role on the expression of PNO1 in lung adenocarcinoma (Figure 1D). PNO1 expression was significantly higher in both Tp53 mutant and wild‐type lung adenocarcinoma compared to normal lung tissues. However, lung adenocarcinoma expressing mutant Tp53 showed significantly higher PNO1 expression than those expressing wild‐type Tp53. We next measured the expression of PNO1 in both male and female lung adenocarcinoma patients (Figure 1E). PNO1 expression was significantly higher in both male and female lung adenocarcinoma patients than those in normal lung tissues. However, lung adenocarcinoma from male patients showed significantly higher PNO1 expression than those from female patients. We next compared the expression of PNO1 among smokers with lung adenocarcinoma (Figure 1F). Lung adenocarcinoma patients were classified into 4 groups; non‐smokers, smokers, reformed smokers (<15 years), and reformed smoker (>15 years). PNO1 expression was higher in lung adenocarcinoma tissues compared to normal tissues. Smokers with lung cancer and reformed smokers (>15 years) showed significantly higher expression of PNO1 than non‐smokers and reformed smokers (<15 years). These data suggest that constant smoking for more than 15 years can have significant effects on PNO1 expression.

### 3.2 PNO1/CRISPR/Cas9 inhibits colony formation and cell viability, and induces apoptosis in lung cancer cells

**FIGURE 2:**
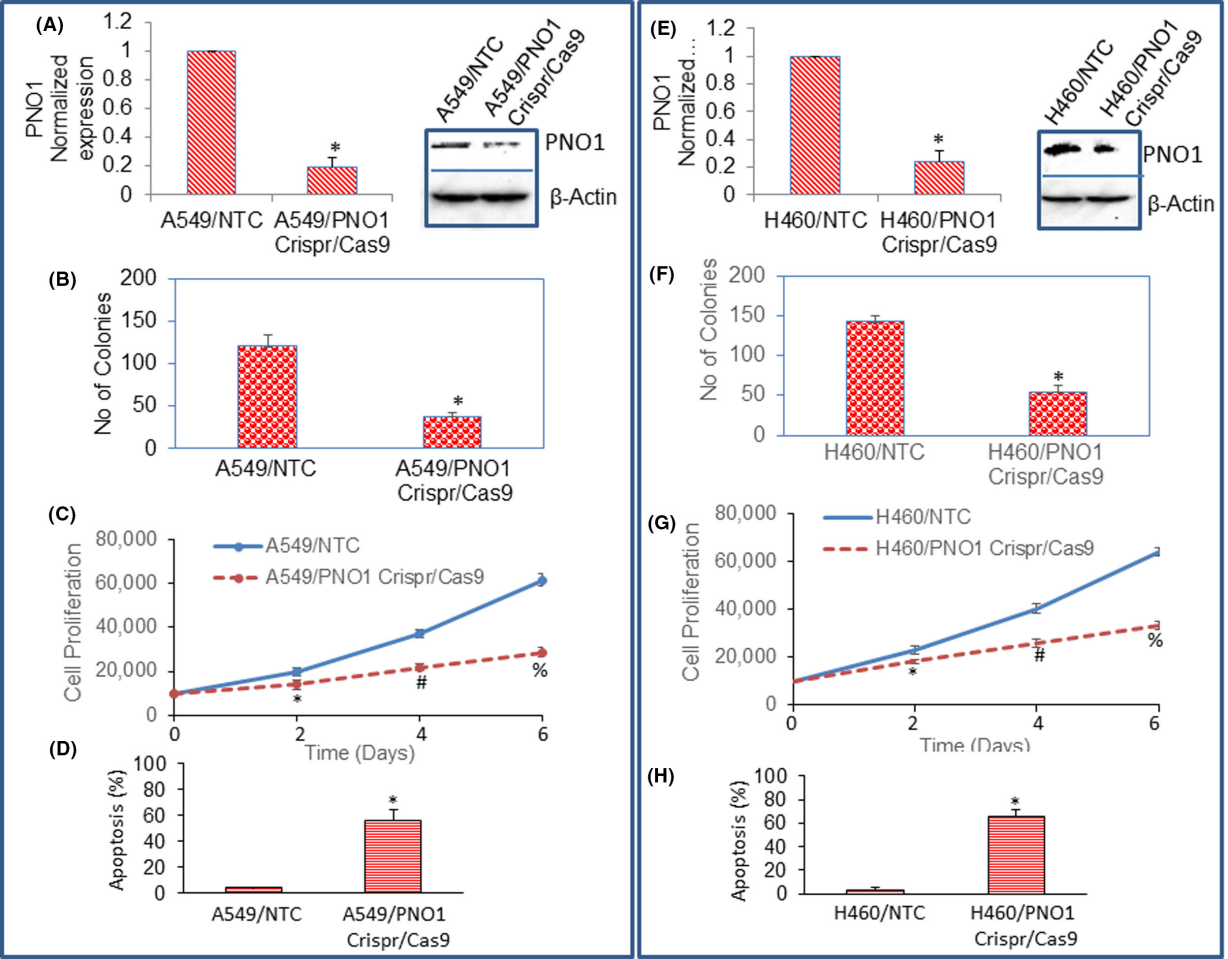
PNO1/CRISPR/Cas9 inhibits colony formation and cell proliferation, and induces apoptosis in lung cancer cells. (A, E) mRNA and protein expression of PNO1. Lung cancer (A549 and H460) cells were infected with lentiviral particles expressing either non‐targeting control (NTC) or PNO1/CRISPR/Cas9, and the mRNA and protein expression of PNO1 was measured by the qRT‐PCR (left) and Western blot analysis (right), respectively. β‐Actin was used as a loading control. (B, F) Colony formation. Lung cancer (A549 and H460) cells were infected with lentiviral particles expressing either NTC or PNO1/CRISPR/Cas9. The number of colonies formed at 21 days were counted. Data represent mean (*n* = 4) ± SD. * = significantly different from NTC, *p* < 0.05. (C, G) Cell viability. A549 and H460 cells were infected with lentiviral particles expressing either NTC or PNO1/CRISPR/Cas9. Cell viability was measured as described in Materials and Methods. Data represent mean (*n* = 4) ± SD. * = significantly different from NTC, *p* < 0.05. (D, H) Apoptosis. A549 and H460 cells were infected with lentiviral particles expressing either NTC or PNO1/CRISPR/Cas9. Apoptosis was measured as described in Materials and Methods. Data represent mean (*n* = 4) ± SD. * = significantly different from NTC, *p* < 0.05.

Since PNO1 expression has been associated with lung cancer progression and poor survival,^8^, ^9^, ^38^ we examined the effects of PNO1 inhibition on lung cancer growth. The PNO1 expression was inhibited by CRISPR/Cas9 technique. A549 and H460 cells were infected with lentiviral particles expressing either NTC or PNO1/CRISPR/Cas9, and the expression of PNO1 mRNA and protein was measured by the qRT‐PCR and Western blot analysis. PNO1/CRISPR/Cas9 inhibited the expression of both PNO1 mRNA and protein in A549 and H460 cells compared to NTC (Figure 2A,E). These data suggest that CRISPR/Cas9 technique is efficient in inhibiting the expression of PNO1 in lung adenocarcinoma.

Colony formation and cell proliferation assays are generally used to assess the effects of CRISPR/Cas9 on cancer cells. Since PNO1/CRISPR/Cas9 inhibited the expression of PNO1, we next sought to examine the effects of inhibiting PNO1 on colony formation by lung cancer cells. The number of colonies formed was counted on day 21. PNO1/CRISPR/Cas9 significantly inhibited colony formation in both A549 and H460 cells (Figure 2B,F). We next sought to examine the effects of inhibiting PNO1 on lung cancer (A549 and H460) cell proliferation (Figure 2C,G). PNO1/CRISPR/Cas9 significantly inhibited cell proliferation of both A549 and H460 cells at 2, 4, and 6 days.

Since PNO1/CRISPR/Cas9 inhibited cell proliferation in both A549 and H460 cells, we next sought to examine the effects of inhibiting PNO1 expression on lung cancer cell apoptosis (Figure 2D,H). PNO1/CRISPR/Cas9 significantly induced apoptosis in both A549 and H460 cells compared to NTC control. These data suggest that PNO1 can be a therapeutic target for lung cancer.

### 3.3 PNO1/CRISPR/Cas9 inhibits cell motility, migration, and invasion in lung adenocarcinoma

**FIGURE 3:**
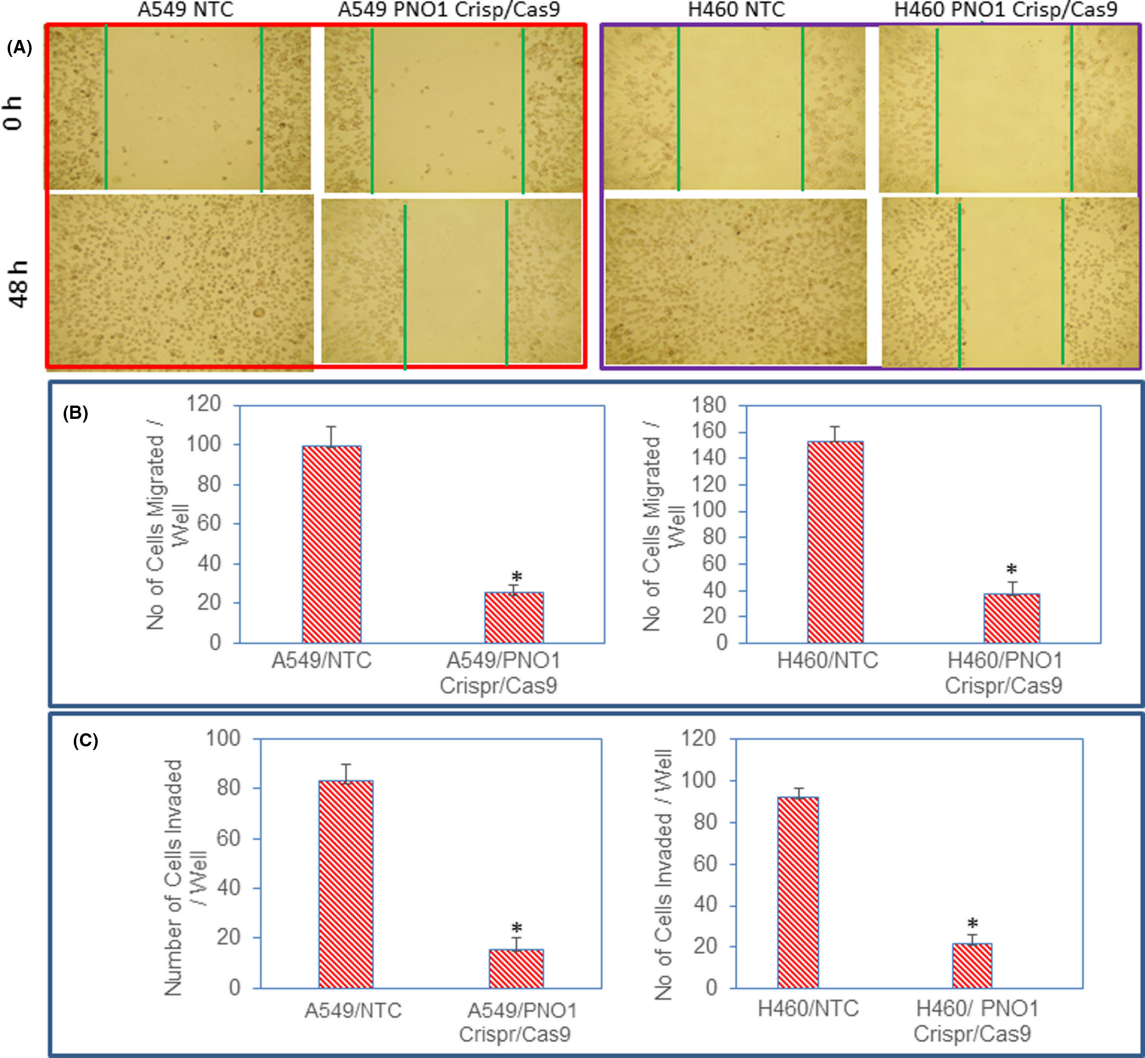
PNO1/CRISPR/Cas9 inhibits cell motility, migration, and invasion. (A) Cell motility assay. Lung cancer cells (A549 and H460) expressing either non‐targeting control (NTC) or PNO1/CRISPR/Cas9 were grown in monolayer and scratched. Cells were photographed at 0 and 48 h. (B) Cell Migration assay. Lung cancer cells (A549 and H460) expressing either NTC or PNO1/CRISPR/Cas9 were seeded. After 48 h of seeding, cell migration assays were performed as described in Materials and Methods. Data represent mean (*n* = 4) ± SD. * = significantly different from control, and each other, *p* < 0.05. (C) Cell invasion assay. Lung cancer cells (A549 and H460) expressing either NTC or PNO1/CRISPR/Cas9 were seeded. After 48 h of seeding, cell invasion assays were performed as described in Materials and Methods. Data represent mean (*n* = 4) ± SD. * = significantly different from control, and each other, *p* < 0.05.

Epithelial–mesenchymal transition (EMT) is a biological process that converts epithelial cells into mesenchymal cells.^39^, ^40^ During EMT, cells undergo genetic changes that allow them to lose polarity, leave the primary site and migrate to a distant location (secondary site) to reestablish, differentiate, proliferate, and survive.^41^ We next measured the effects of PNO1/CRISPR/Cas9 on cell motility. PNO1/CRISPR/Cas9 inhibited cell motility of both A549 and H460 cells (Figure 3A). Since inhibition of PNO1 suppressed cell motility, we next measured the effects of PNO1/CRISPR/Cas9 on cell migration and invasion. PNO1/CRISPR/Cas9 inhibited cell migration and invasion of both A549 and H460 cells (Figure 3B,C). These data suggest that inhibition of PNO1 can be beneficial for suppressing lung cancer cell metastasis.

### 3.4 PNO1/CRISPR/Cas9 regulates the expression of markers of epithelial–mesenchymal transition in lung adenocarcinoma

**FIGURE 4:**
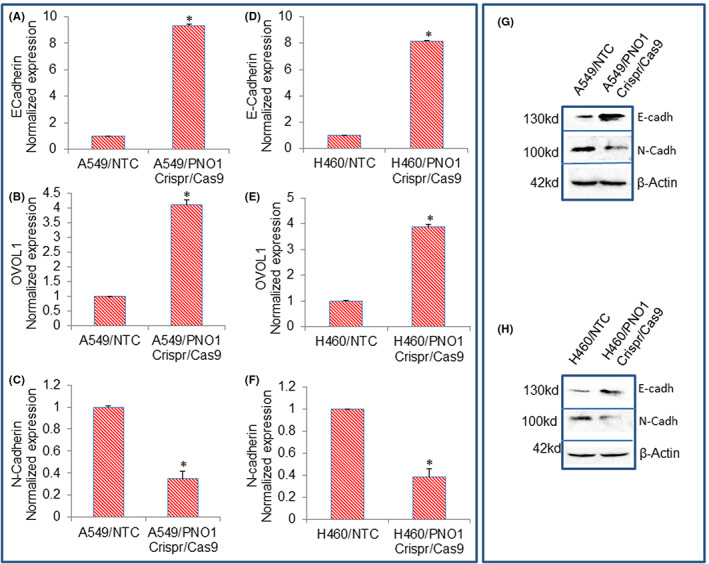
Effects of PNO1/CRISPR/Cas9 on the expression of E‐Cadherin, OVOL1, and N‐Cadherin in lung cancer cells. (A–C) Lung cancer A549 cells expressing either non‐targeting control (NTC) or PNO1/CRISPR/Cas9 were seeded. After 24 h, cells were harvested, and RNA was extracted to measure the mRNA expression of E‐cadherin, OVOL1, and N‐Cadherin by qRT‐PCR. Data represent mean ± SD. * = significantly different from control, *p* < 0.05. (D–F) Lung cancer H460 cells expressing either NTC or PNO1/CRISPR/Cas9 were seeded. After 24 h, cells were harvested, and RNA was extracted to measure the mRNA expression of E‐cadherin, OVOL1, and N‐Cadherin by qRT‐PCR. Data represent mean ± SD. * = significantly different from control, *p* < 0.05. (G) Protein expression of cadherins in A549 cells. Cell lysates were collected from A549/NTC and A549/PNO1/CRISPR/Cas9 cells and protein expression of E‐cadherin and N‐cadherin was measured by the Western blot analysis. (H) Protein expression of cadherins in H460 cells. Cell lysates were collected from H460/NTC and H460/PNO1/CRISPR/Cas9 cells and protein expression of E‐cadherin and N‐Cadherin was measured by the Western blot analysis. β‐Actin was used as a loading control.

Epithelial–mesenchymal transition (EMT) occurring during tumour progression is highly deregulated.^41^ Transcription factors such as Snail, Slug, and ZEB1 are involved in the orchestration of EMT.^41^, ^42^ Since PNO1 knockdown inhibited cell motility, migration, and invasion, we next examined the molecular mechanisms of EMT regulation by measuring the expression of epithelial (E‐cadherin and OVOL1) and mesenchymal markers (N‐cadherin). PNO1/CRISPR/Cas9 induced the mRNA expression of E‐cadherin and OVOL1 and inhibited the mRNA expression of N‐cadherin (Figure 4A–F). We next confirmed the protein expression of E‐ and N‐cadherins by the Western blot analysis. PNO1/CRISPR/Cas9 induced the protein expression of E‐cadherin and inhibited the protein expression of N‐cadherin (Figure 4G,H).

**FIGURE 5:**
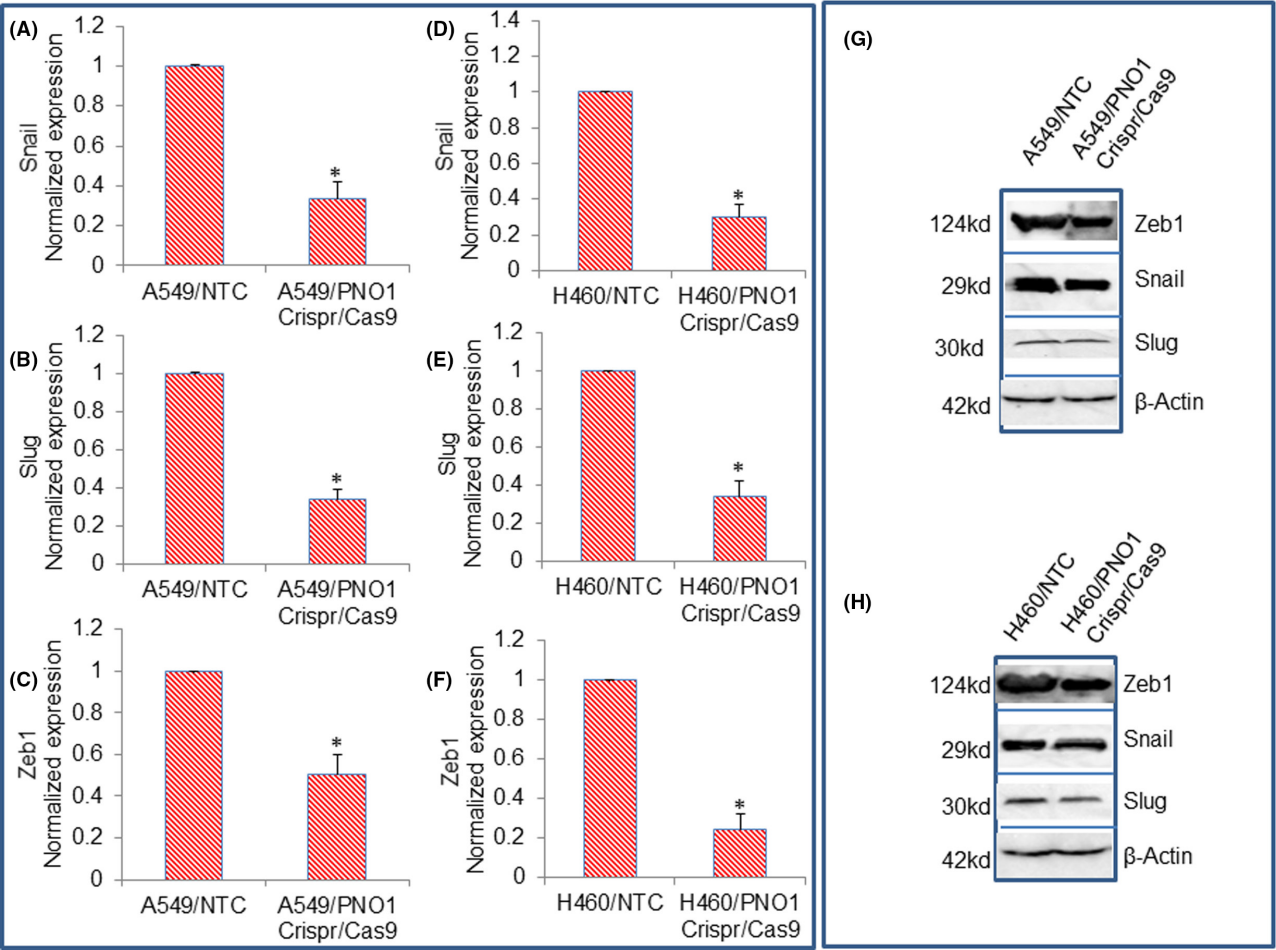
Effects of PNO1/CRISPR/Cas9 on the expression of EMT‐related transcription factors in lung cancer cells. (A–C) Lung cancer A549 cells expressing either non‐targeting control (NTC) or PNO1/CRISPR/Cas9 were seeded. After 24 h, cells were harvested, and RNA was extracted to measure the expression of Snail, Slug, and Zeb1 by qRT‐PCR. Data represent mean ± SD. * = significantly different from control, *p* < 0.05. (D–F) Lung cancer H460 cells expressing either NTC or PNO1/CRISPR/Cas9 were seeded. After 24 h, cells were harvested, and RNA was extracted to measure the expression of Snail, Slug, and Zeb1 by qRT‐PCR. Data represent mean ± SD. * = significantly different from control, *p* < 0.05. (G) Protein expression of Snail, Slug, and Zeb1 in A549 cells. Cell lysates were collected from A549/NTC and A549/PNO1/CRISPR/Cas9 cells and protein expression of Snail, Slug, and Zeb1 was measured by the Western blot analysis. (H) Protein expression of Snail, Slug, and Zeb1 in H460 cells. Cell lysates were collected from H460/NTC and H460/PNO1/CRISPR/Cas9 cells and protein expression of Snail, Slug, and Zeb1 were measured by the Western blot analysis. β‐Actin was used as a loading control.

Expression of epithelial and mesenchymal markers is controlled by EMT transcription factors.^40^, ^43^ Since PNO1 knockdown modulated the expression of E‐cadherin, OVOL1, and N‐cadherin, we next sought to measure the mRNA and protein expression of Snail, Slug, and Zeb1. As shown in Figure 5A–H, PNO1/CRISPR/Cas9 inhibited the mRNA and protein expression of Snail, Slug, and Zeb1 in lung cancer A549 and H460 cells. These data suggest that PNO1 knockdown can inhibit EMT by modulating the expression of cadherins, OVOL1, and transcription factors Snail, Slug, and Zeb1.

### 3.5 PNO1/CRISPR/Cas9 regulates genes involved in the cell cycle and inflammation in lung cancer cells

**FIGURE 6:**
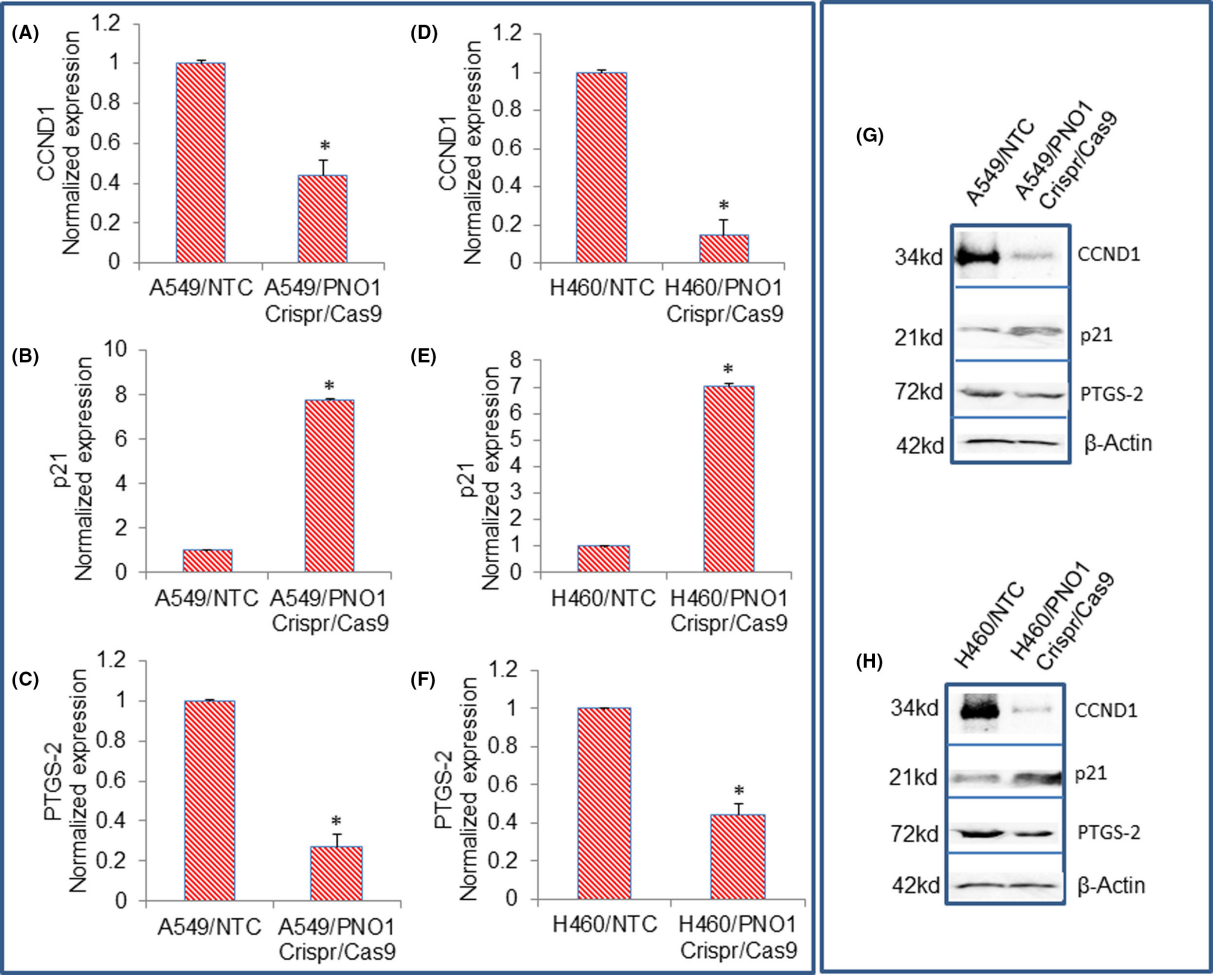
Effects of PNO1/CRISPR/Cas9 on the expression of CCND1, p21, and PTGS‐2 in lung cancer cells. (A–C) Lung cancer A549 cells expressing either non‐targeting control (NTC) or PNO1/CRISPR/Cas9 were seeded. After 24 h, cells were harvested, and RNA was extracted to measure the expression of CCND1, p21, and PTGS‐2 by qRT‐PCR. Data represent mean ± SD. * = significantly different from control, *p* < 0.05. (D–F) Lung cancer H460 cells expressing either NTC or PNO1/CRISPR/Cas9 were seeded. After 24 h, cells were harvested, and RNA was extracted to measure the expression of CCND1, p21, and PTGS‐2 by qRT‐PCR. Data represent mean ± SD. * = significantly different from control, *p* < 0.05. (G) Protein expression of CCND1, p21, and PTGS‐2 in A549 cells. Cell lysates were collected from A549/NTC and A549/PNO1/CRISPR/Cas9 cells and protein expression of CCND1, p21, and PTGS‐2 were measured by the Western blot analysis. (H) Protein expression of CCND1, p21, and PTGS‐2 in H460 cells. Cell lysates were collected from H460/NTC and H460/PNO1/CRISPR/Cas9 cells and protein expression of CCND1, p21, and PTGS‐2 were measured by the Western blot analysis. β‐Actin was used as a loading control.

Activation or overexpression of oncogenes enhances cell division because most cell cycle regulator proteins are the products of oncogenes.^44^, ^45^ CCND1 gene acts at the G1/S stage of the cell cycle and is frequently overexpressed in cancer.^46^, ^47^, ^48^ P21 inhibits cell cycle progressions at G1/S and G2/M transition.^46^, ^47^, ^48^ We, therefore, examined the effects of PNO1 knockdown on the mRNA and protein expression of CCND1 and p21. PNO1/CRISPR/Cas9 inhibited the mRNA and protein expression of CCND1 and upregulated the mRNA and protein expression of cell cycle inhibitor p21 in A549 and H460 cells (Figure 6A,B,D,E,G,H). These data suggest that PNO1 can regulate the cell cycle in lung cancer.

PTGS2 is dynamically regulated during the initiation and resolution of acute inflammation. PTGS2 plays a role in the prostanoid synthesis involved in the initiation of inflammation and mitogenesis. We, therefore, examined the effects of PNO1 knockdown on the mRNA and protein expression PTGS2. PNO1/CRISPR/Cas9 inhibited the mRNA and protein expression of PTGS2 in A549 and H460 cells (Figure 6C,F–H). These data suggest that inhibition of PNO1 can suppress inflammation in lung adenocarcinoma.

**FIGURE 7:**
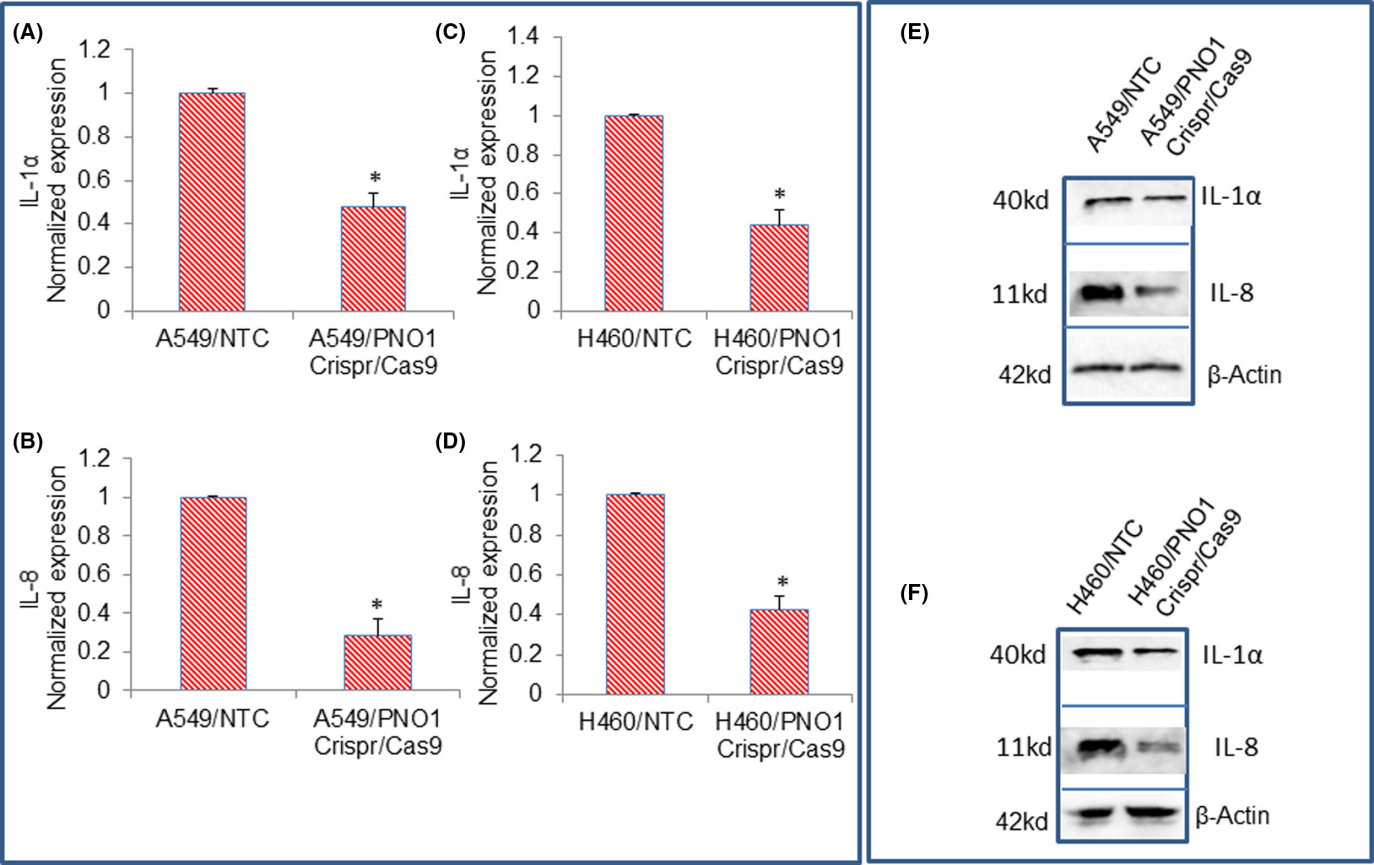
Effects of PNO1/CRISPR/Cas9 on the expression of IL‐1α, IL‐8, and CXCL‐8 in lung cancer cells. (A–C) Lung cancer A549 cells expressing either non‐targeting control (NTC) or PNO1/CRISPR/Cas9 were seeded. After 24 h, cells were harvested, and RNA was extracted to measure the mRNA expression of IL‐1α, and IL‐8 by qRT‐PCR. Data represent mean ± SD. * = significantly different from control, *p* < 0.05. (D–F) Lung cancer H460 cells expressing either NTC or PNO1/CRISPR/Cas9 were seeded. After 24 h, cells were harvested, and RNA was extracted to measure the mRNA expression of IL‐1α, and IL‐8 by qRT‐PCR. Data represent mean ± SD. * = significantly different from control, *p* < 0.05. (E) Protein expression of IL‐1α, and IL‐8 in A549 cells. Cell lysates were collected from A549/NTC and A549/PNO1/CRISPR/Cas9 cells and protein expression of IL‐1α, and IL‐8 was measured by the Western blot analysis. (F) Protein expression of IL‐1α, and IL‐8 in H460 cells. Cell lysates were collected from H460/NTC and H460/PNO1/CRISPR/Cas9 cells and protein expression of IL‐1α, and IL‐8 was measured by the Western blot analysis. β‐Actin was used as a loading control.

Since PNO1 knockdown inhibited the expression of PTGS2, a gene involved in inflammation, we sought to examine the effects of inhibiting PNO1 on the expression of inflammatory cytokines (IL‐1α and IL‐8) in lung cancer cells. PNO1/CRISPR/Cas9 inhibited the mRNA and protein expression of IL‐1α, and IL‐8 in A549 and H460 cells (Figure 7A–F). These data suggest that PNO1 knockdown may inhibit inflammation by suppressing inflammatory cytokines in lung adenocarcinoma.

### 3.6 PNO1/CRISPR/Cas9 inhibits Notch signalling pathway and targets lung cancer cells

**FIGURE 8:**
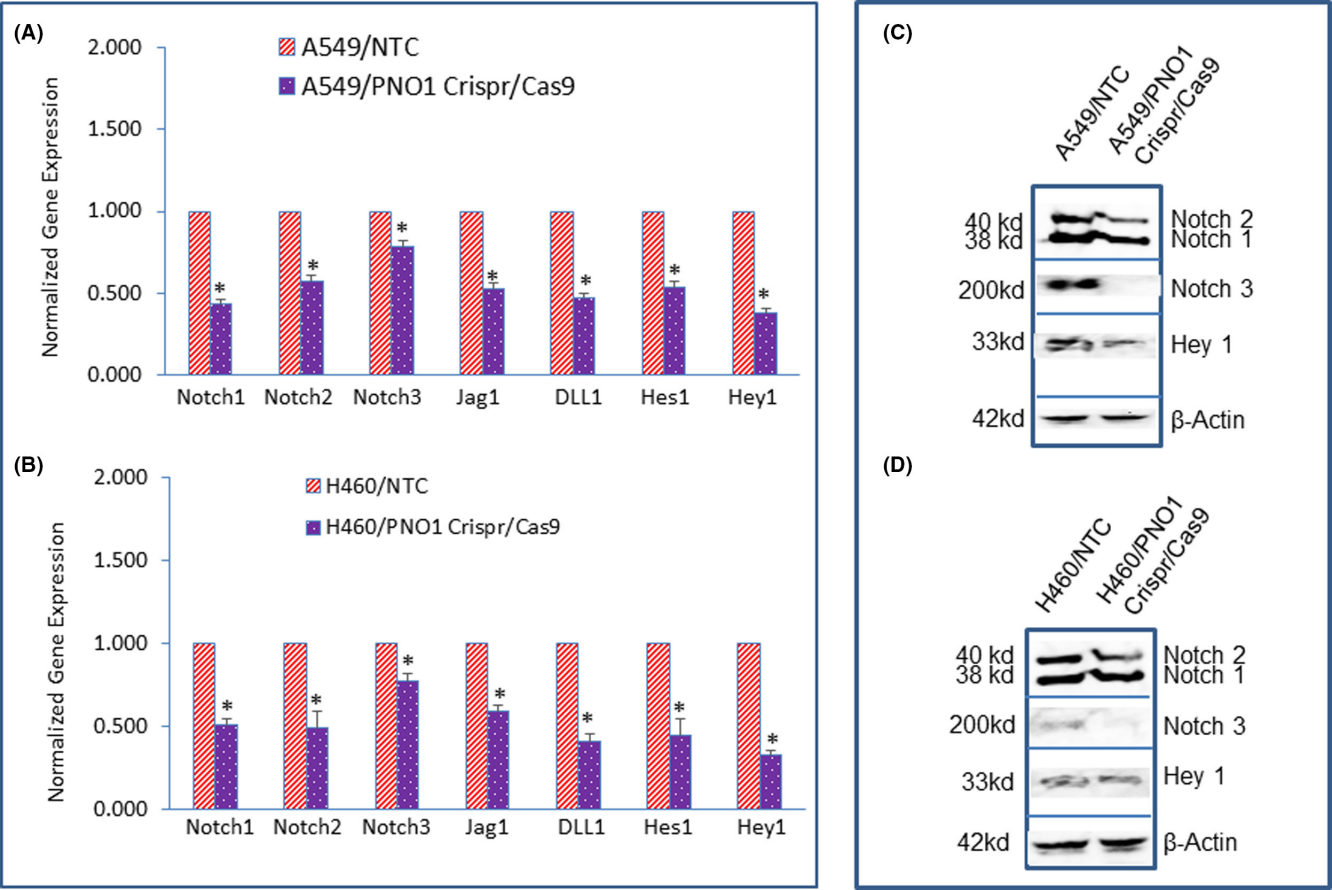
Effects of PNO1/CRISPR/Cas9 on Notch signalling pathway in lung cancer cells. (A) Lung cancer A549 cells expressing either non‐targeting control (NTC) or PNO1/CRISPR/Cas9 were seeded. After 24 h, cells were harvested, and RNA was extracted to measure the mRNA expression of Notch1, Notch2, Notch3, Jagged1, DLL1, Hes1, and Hey1 by qRT‐PCR. Data represent mean ± SD. * = significantly different from control, *p* < 0.05. (B) Lung cancer H460 cells expressing either NTC or PNO1/CRISPR/Cas9 were seeded. After 24 h, cells were harvested, and RNA was extracted to measure the mRNA expression of Notch1, Notch2, Notch3, Jagged1, DLL1, Hes1, and Hey1 by qRT‐PCR. Data represent mean ± SD. * = significantly different from control, *p* < 0.05. (C) Protein expression of Notch1, 2, 3, and Hey1 in A549 cells. Cell lysates were collected from A549/NTC and A549/PNO1/CRISPR/Cas9 cells and protein expression of Notch1, 2, 3, and Hey1 was measured by the Western blot analysis. (D) Protein expression of Notch1, 2, 3, and Hey1 in H460 cells. Cell lysates were collected from H460/NTC and H460/PNO1/CRISPR/Cas9 cells and protein expression of Notch1, 2, 3, and Hey1 was measured by the Western blot analysis. β‐Actin was used as a loading control.

Since the Notch signalling pathway plays a crucial role in lung carcinogenesis,^25^, ^49^ we sought to measure the effects of PNO1 knockdown on the components of the Notch pathway and its target genes. PNO1/CRISPR/Cas9 inhibited the expression of Notch1, Notch2, Notch3 Jagged1, and DLL1 in lung cancer A549 and H460 cells (Figure 8A,B). Similarly, PNO1/CRISPR/Cas9 inhibited the expression of Notch target genes Hes1 and Hey1 in lung cancer A549 and H460 cells (Figure 8A,B). We next confirmed the effects of PNO1 knockdown on the expression of some of the proteins of the Notch pathway. PNO1/CRISPR/Cas9 inhibited the protein expression of Notch1, Notch2, Notch3, and Hey1 in both A549 and H460 cell lines (Figure 8C,D). These data suggest that PNO1 knockdown can inhibit lung carcinogenesis by targeting the Notch signalling pathway.

## 4 DISCUSSION

Despite extensive research on lung adenocarcinoma, clinical outcomes remain very poor. Therefore, novel therapeutic technologies are urgently needed for the management of the disease. Considering the role of genetics and epigenetics in carcinogenesis, gene therapy provides an attractive approach in cancer treatment research. Gene therapy causes fewer side effects to patients compared to conventional methods such as chemotherapy and radiotherapy. Furthermore, the gene therapy approach offers a persistent cure compared to traditional therapy which generally ends up in drug resistance and relapse. PNO1/CRISPR/Cas9 technique could be an effective strategy for genome editing and thus treating patients.^50^ This system comprises Cas9 (RNA‐guided DNA endonuclease) and gRNA. Specifically, this system has been used to alter site‐specific mutagenesis, gene expression, and epigenetics and to target RNAs and specific DNA sequences.^51^, ^52^ In the present study, inhibition of PNO1 expression by the CRISPR/Cas9 technique suppressed lung adenocarcinoma. PNO1 knockdown inhibited lung cancer cell viability, colony formation, and EMT, and induced apoptosis. In addition to inhibiting the growth of lung adenocarcinoma, PNO1 knockdown also inhibited Notch signalling pathway, and cytokines and chemokines. PNO1/CRISPR/Cas9 inhibited EMT by inducing a cadherin switch, up‐regulating OVOL1, and suppressing the expression of EMT transcription factors (Snail. Slug and Zeb1). Our findings suggest that PNO1 knockdown can be a beneficial therapeutic agent for the treatment of lung adenocarcinoma. Clinical trials are needed to demonstrate the safety and efficacy of PNO1/CRISPR/Cas9.

Cancer cells rely on ribosome biogenesis which increases in cancer cells to cope with a rise in protein synthesis and sustain unrestricted growth.^53^, ^54^ The oncogenic role of PNO1 in cancer has recently been reported.^7^, ^8^, ^9^, ^11^, ^38^, ^55^ In hepatocellular carcinoma, celecoxib inhibited PNO1 expression and tumour growth through modulation of AKT/mTOR signalling pathway.^7^ In colorectal cancer, EBF1 over‐expression down‐regulated PNO1 expression and transcription, and up‐regulated the expression of p53 and p21 proteins.^10^ In lung adenocarcinoma, higher expression of PNO1 has been associated with poor survival.^9^ According to TCGA data, PNO1 expression in lung adenocarcinoma patients increased with stage of development, nodal metastasis, and smoking. Lung adenocarcinoma tissues from males expressed higher PNO1 than those from females, suggesting the influence of sex on PNO1 expression. Furthermore, lung adenocarcinoma tissues with mutant Tp53 expressed higher PNO1 than those with wild‐type Tp53, suggesting the influence of Tp53 status on PNO1 expression. In the present study, PNO1 was overexpressed in lung adenocarcinoma, and its inhibition by CRISPR/Cas9 technology inhibited cell proliferation, motility, migration, and invasion, and induced apoptosis. Our data are in agreement with others where PNO1 has been shown to promote cell proliferation and migration, and PNO1 knockdown inhibited tumorigenesis.^7^, ^8^, ^10^, ^11^, ^38^ These data suggest that PNO1 can be used as a diagnostic and prognostic biomarker for lung cancer, and its inhibition can be used for the treatment of cancer.

The NOTCH signalling pathway plays a crucial role in lung development, growth, differentiation, and tissue regeneration.^25^, ^49^ Improper activation of the NOTCH pathway has been associated with lung adenocarcinoma.^25^, ^49^ In the present study, we have demonstrated that PNO1 knockdown inhibited Notch signalling by suppressing the expression of Notch receptors (Notch1, Notch2, and Notch3), their ligands (Jagged 1 and DLL1), and downstream targets Hes‐1 and Hey1. Similarly, another study has demonstrated the oncogenic role of PNO1 where PNO1 promoted lung adenocarcinoma progression through the Notch signalling pathway.^9^ Overall, these data suggest that PNO1 is an oncogenic factor, and its inhibition by CRISPR/Cas9 can suppress lung carcinogenesis by modulating the Notch pathway. The approach of targeting the NOTCH signalling pathway represents a promising therapeutic strategy for lung cancer.

The process of EMT during carcinogenesis is highly deregulated.^40^, ^43^ During EMT, cancer cells undergo genetic changes, become motile, nonpolarized, and invasive while maintaining their primary tumour characteristics. Mesenchymal–epithelial transition, a reverse of EMT, is characterized by the colonization of malignant cells at the secondary/distant sites.^40^, ^43^ Therefore, EMT modulation could constitute an approach to avoid metastasis. Transcription factors such as Twist1, Snail, Slug, and ZEB1 are involved in the modulation of EMT. During EMT, the expression of mesenchymal markers such as vimentin and N‐cadherin is increased and the expression of epithelial markers such as E‐cadherin is decreased. The loss of E‐cadherin expression has been shown as an unfavourable prognostic factor in non‐small cell lung cancer (NSCLC).^56^, ^57^ In support of this concept, the expression of Vimentin and Snail has also been linked with the malignant phenotype of NSCLC.^56^, ^57^ During carcinogenesis, Snail generally acts as an inducer, while Twist and Zeb ½ are principally involved in retaining the invasive mesenchymal phenotype.^58^ In the present study, PNO1 knockdown inhibited EMT by inducing a cadherin switch, inducing OVAL1 and inhibiting Snail, Slug, and Zeb1, and indicating the significance of PNO1 knockdown for suppressing EMT and metastasis.

A direct link between ribosome biogenesis and cell cycle regulation has been reported. Impaired ribosome biogenesis induces a checkpoint control that prevents cell cycle progression.^59^ In another study, the p53 pathway as a mediator (p53‐dependent) of the signalling link between ribosome biogenesis and the cell cycle was demonstrated.^60^ Transgenic mice model overexpressing Myc has demonstrated the importance of p53 in the inhibition of cell proliferation in response to obstructed ribosome biogenesis.^61^ Downregulation of ribosome biogenesis by haploinsufficiency reduces cell proliferation and extends tumour formation in p53 wild‐type but not in p53 null mice. Inhibition of ribosome biogenesis suppresses cell proliferation by blocking the G_1_/S phase transition through the p21‐mediated suppression of pRb phosphorylation.^60^ Interestingly, the suppression of ribosome biogenesis resulted in cell cycle arrest in a p53‐independent manner.^62^, ^63^ In the present study, inhibition of PNO1‐induced p21 and suppressed CCND1 in lung cancer cells. Overall, these data links ribosome biogenesis and cell cycle regulation in both p53‐dependent and independent manners.

Chronic inflammation has been linked with an increased rate of ribosome biogenesis.^64^, ^65^ During inflammation, elevated levels of prostaglandins, inflammatory cytokines, and chemokines have been observed.^66^, ^67^, ^68^, ^69^ These inflammatory signals are sufficient to trigger cancer initiation. In the present study, inhibition of PNO1 downregulated PTGS‐2, IL‐1α, IL‐8, and CXCL‐8 in A549 and H460 cells. Our data suggest that the inhibition of PNO1 can inhibit lung adenocarcinoma by suppressing the production of inflammatory cytokines and chemokines.

In conclusion, our study has demonstrated that inhibition of PNO1 expression can inhibit lung cancer growth and EMT by suppressing the NOTCH pathway. Further studies are needed to examine the clinical potential of PNO1 inhibition in lung adenocarcinoma. Therefore, PNO1 inhibition by genetic or pharmacological means offers new hope for the treatment of lung adenocarcinoma.

## AUTHOR CONTRIBUTIONS

**Sanjit K Roy:** Conceptualization (equal); data curation (equal); formal analysis (equal); investigation (equal); methodology (equal); project administration (equal); validation (equal); visualization (equal); writing – original draft (equal); writing – review and editing (equal). **Shivam Srivastava:** Formal analysis (equal); methodology (equal); validation (equal); visualization (equal). **Andrew Hancock:** Conceptualization (equal); formal analysis (equal); methodology (equal); project administration (equal); validation (equal); visualization (equal); writing – original draft (equal). **Anju Shrivastava:** Visualization (equal); writing – review and editing (equal). **Jason Morvant:** Visualization (equal); writing – review and editing (equal). **Sharmila Shankar:** Methodology (equal); resources (equal); supervision (equal); visualization (equal); writing – review and editing (equal). **Rakesh K. Srivastava:** Resources (equal); supervision (equal); visualization (equal); writing – review and editing (equal).

## CONFLICTS OF INTEREST

All the authors have declared that no competing interests exist.

## Data Availability

The data that support the findings of this study are available from the corresponding author upon reasonable request.
